# Retroperitoneal fasciae as barriers for nerve and arterial passages connecting the retroperitoneal region to the peritoneal organs

**DOI:** 10.1111/joa.14036

**Published:** 2024-03-07

**Authors:** Yuzuki Sugiyama, Satoru Muro, Daisuke Ban, Keiichi Akita

**Affiliations:** ^1^ Department of Clinical Anatomy Tokyo Medical and Dental University (TMDU) Tokyo Japan; ^2^ Department of Hepatobiliary and Pancreatic Surgery National Cancer Center Hospital Tokyo Japan

**Keywords:** celiac plexus, pancreas, pancreatic head plexus, retropancreatic fascia of Toldt, retropancreatic fascia of Treitz

## Abstract

The fascia of the pancreatic head is referred to as the retropancreatic fascia of Treitz, and that of the body and tail of the pancreas is named the retropancreatic fascia of Toldt. However, the spatial relationship between the nerves, fascia, and the distribution of the fascia on the dorsal side of the pancreas remains unclear. Therefore, this study aimed to explore the distribution of these fasciae and elucidate the spatial relationship between the nerves and arteries connecting the retroperitoneal space and the peritoneal organs by studying eight cadavers using macroscopic anatomical examination, wide‐range serial sectioning, and three‐dimensional reconstruction. The fasciae of Treitz and Toldt converge caudally to the root of the superior mesenteric artery (SMA), forming a narrower gap around the roots of the celiac trunk and SMA than in the celiac plexus. The fasciae eventually get closer to each other, and the boundary between them becomes obscured, providing coverage to the anterior surface of the aorta between the SMA and the inferior mesenteric artery. The celiac plexus does not penetrate the fascia but converges before spreading into the pancreas. Similarly, the arteries pass through this gap in the fasciae. Our findings suggest that the retroperitoneal space and peritoneal organs are connected through a narrow no‐fascia area, with the distribution of the fascia relating to nervous and vascular pathways. Our findings reveal that the distribution of the avascular plane may provide a crucial anatomical foundation for abdominal digestive organ surgery by reducing bleeding volume and determining the dissection region.

## Graphical abstract

The right and left side fasciae converge and cover the aorta's anterior surface between the superior and inferior mesenteric artery roots. The celiac plexus converges within a slender gap within the fascia around the root of the celiac trunk and superior mesenteric artery. Our study underscores that the retroperitoneal space and peritoneal organs are bridged solely by a narrow gap between the celiac trunk and superior mesenteric artery, along with the nerves enveloping these arteries.

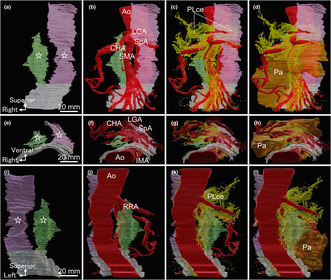

## 1 INTRODUCTION

Surgical exploration has traditionally focused on aspects such as blood circulation and organ innervation. However, recent advancements in laparoscopic and robotic surgery, offering high‐magnification and high‐resolution images, have drawn attention towards membranous structures within the body. Noteworthy among these structures are the fascia, mesentery, and peritoneum. “Fasciae of fusion,” a significant component, results from the fusion of embryonic mesentery with the body wall (Congdon et al., 1942; Toldt, 1893). Functioning as an avascular plane, it serves as both a dissection plane during surgery and a key reference point for mobilisation (Cattell & Braasch, 1960; Heald et al., 1982; Hohenberger et al., 2009; Kocher., 1903; Shinohara et al., 2018). Additionally, it plays a critical role in impeding cancer cell infiltration (Kitagawa et al., 2013). Remarkably, even after the fusion, the mesentery's continuity remains intact, prompting advocacy for considering the mesentery as an independent organ (Byrnes et al., 2021; Coffey et al., 2022; Coffey & O'Leary, 2016). Thus, a broader and in‐depth understanding of mesenteric anatomy holds substantial implications for surgical procedures.

Particularly, the fascia situated dorsal to the pancreatic head is referred to as the “retropancreatic fascia of Treitz,” whereas the fascia enveloping the body and tail of the pancreas is termed the “retropancreatic fascia of Toldt” (Toldt, 1879; Treitz, 1853). These fasciae are primarily composed of loose connectives, distinguishing them from the peritoneum in terms of the histological and immunohistological features (Yang et al., 2013). Furthermore, the region around the pancreas contains a dense distribution of nerve fibres, known as the mesopancreas and pancreatic head plexus, originating from the celiac plexus. Despite radiating radially in a plate‐like structure, these nerve fibres remain confined within the fascia. (Gockel et al., 2007; Muro et al., 2021; Yoshioka & Wakabayashi, 1958). These findings suggest a complex relationship between the fascia and nerve distribution.

To the best of our knowledge, a comprehensive and detailed understanding of the fascia distribution on the dorsal side of the pancreas is lacking due to the challenges posed by the discontinuity of mesothelial cells. Additionally, previous anatomical analyses of pancreatic nerves required the removal of the fascia to dissect the nerves (Muro et al., 2021; Nagakawa et al., 2020; Yi et al., 2003; Yi et al., 2020). Although these studies have clarified the nerve distribution on the ventral side of the fascia, the spatial relationship between the nerves and fascia, including the distribution on the dorsal side, remains unclear. The fascia of Treitz generally serves as the dissection plane in both the Kocher and Cattell–Braasch manoeuvres performed during pancreaticoduodenectomy (Cattell & Braasch, 1960; Kocher., 1903). The Kocher manoeuvre involves detaching the first and proximal second parts of the duodenum and the pancreatic head from the inferior vena cava (IVC) and aorta along the fascia, with the superior mesenteric artery (SMA) as mobilisation boundary (Elmslie, 1973; Livani et al., 2022). Similarly, the Cattell–Braasch manoeuvre raises the mesentery of the small intestine and right half of the colon along the fascia to expose the duodenum (Cattell & Braasch, 1960). In addition to the Kocher manoeuvre, the duodenal mobilisation in the Cattell–Braasch manoeuvre involves mobilisation of the third and fourth duodenal parts to the cranial side, allowing for complete visualisation of the SMA origin (Del Chiaro et al., 2015). The dissection plane in the Cattell–Braasch manoeuvre extends beyond the dorsal surface of the pancreatic head, reaching the midline following the avascular plane. Although it can be inferred that the extent of the fascia of Treitz reaches the midline, confirmation is still lacking. Similarly, the precise extent of Toldt's fascia remains uncertain.

Therefore, in this study, we aimed to elucidate the detailed distribution of the fascia on the pancreas's dorsal side, clarify its spatial relationship with the nerves and arteries, and ultimately analyse its anatomical fascia as an avascular plane, which may enhance surgical precision.

## 2 METHODS

### 2.1 Preparation of cadaveric specimens

Eight cadavers (age at death: 88.8 years; age range: 81–94 years; two men and six women) were utilised for this study. Cadavers with a history of upper abdominal surgery were excluded. These cadavers were donated by our department under the guidelines of the Japanese law titled “The Act on Body Donation for Medical and Dental Education” (Act No. 56 of 1983). Before their deaths, all donors voluntarily declared that their bodies would be donated for educational and research purposes. Informed consent was obtained prior to death after explaining the purpose and procedures of using the donor cadavers. Subsequently, the informed consent was reiterated to the donors' relatives upon their passing, with no objections raised. The cadavers underwent fixation via arterial perfusion with 8% formalin and were preserved in 30% alcohol. The study was approved by the Board of Ethics of Tokyo Medical and Dental University (approval number: M2019‐070). All methods were performed in accordance with the relevant guidelines and regulations.

### 2.2 Macroscopic anatomy

For the macroscopic anatomical analysis of the upper abdomen, five cadavers were used. The upper abdomen, including the pancreas, stomach, duodenum, jejunum, liver, spleen, aorta, IVC, and parietal peritoneum, was excised en bloc from the cadavers. The macroscopic examination was conducted using three distinct approaches: duodenal mobilisation to the medial side, duodenal mobilisation to the cranial side, and dissection of the dorsal aspect of the celiac plexus. These techniques were applied to two specimens, two others, and a final specimen.

During the duodenal mobilisation to the medial side, the peritoneum was incised at the right edge of the duodenum, following the Kocher manoeuvre used in pancreaticoduodenectomy. Subsequently, the duodenum and pancreatic head were medially mobilised, and nerve dissection was performed. Similarly, during the duodenal mobilisation to the cranial side, the peritoneum was incised at the inferior border of the duodenum. The dissection of the dorsal aspect of the celiac plexus was performed to explore the upper abdomen from the dorsal perspective to ascertain the inherent positional relationship between the fascia and nerves without invoking mobilisation.

### 2.3 Histology

For the histological analysis of transverse sections, an additional three cadavers were employed. Specimens encompassing the uncinate process, pancreatic, head, neck, and body; aorta, celiac trunk (CeT), SMA, IVC, and celiac plexus were harvested en bloc. These tissue blocks measured 25 mm anteroposteriorly, 60 mm laterally, and 85 mm vertically. Previous work referred to this histological examination method using large tissue masses as “wide‐range serial sectioning” (Muro & Akita, 2023). The harvested tissue blocks were immersed in 10% formalin for 24 h for fixation. Subsequently, a 5‐day decalcification process was conducted using Plank‐Rychlo solution (AlCl3:6H2O 126.7 g/L, HCl 85 mL/L, HCOOH 50 mL/L), followed by neutralisation in a 5% sodium sulphate solution for 12 h. Dehydration steps included sequential immersion in 70, 80, 90, and 100% ethanol (two times) and xylene (two times), with each step taking at least 24 h. The tissue block was then paraffin‐embedded over 5 days under negative pressure, with the paraffin solution changed three times. The paraffin‐embedded tissue blocks were sectioned serially in the transverse plane into 8‐μm thick slices at 0.25‐mm intervals. The whole serial sections were stained with Haematoxylin and eosin to visualise the mesentery, peritoneum, nerves, and other structures. The parts of the serial sections were stained with Masson's trichrome to visualise the collagen fibres to determine the fascia.

### 2.4 Three‐dimensional (3D) reconstruction

Among the three specimens used for histological examination, two were selected for 3D reconstruction. Stained specimens were scanned as whole slides using a high‐quality scanner (GT‐X980; Seiko Epson Corp., Tokyo, Japan). Through microscopic examination, mesentery and parietal peritoneum comprising a simple squamous epithelium were identified in all the slides. The layers of collagen fibres continuous with the folding point of the simple squamous epithelium were identified as fascia. Continuous microscopic examination of slides at 0.25‐mm intervals allowed the identification of fascia in all slides. Using the curve tool in Microsoft PowerPoint, we delineated and coloured various structures, including nerves as yellow, arteries as red, simple squamous epithelium as green, dark green, purple, and dark pink, and fascia comprising layers of collagen fibres as yellow‐green, pink, and white, pancreas as orange, veins as blue and light blue, and lymph nodes as violet. The sequence of the sections was reconstructed using TRI/3D‐ SRFII (ver. R.11.00.00.0‐H. Ratoc, Tokyo, Japan; https://www.ratoc.com/eng/). Further details regarding these methods are described in an earlier study (Muro & Akita, 2023).

## 3 RESULTS

### 3.1 Macroscopic anatomy

**FIGURE 1:**
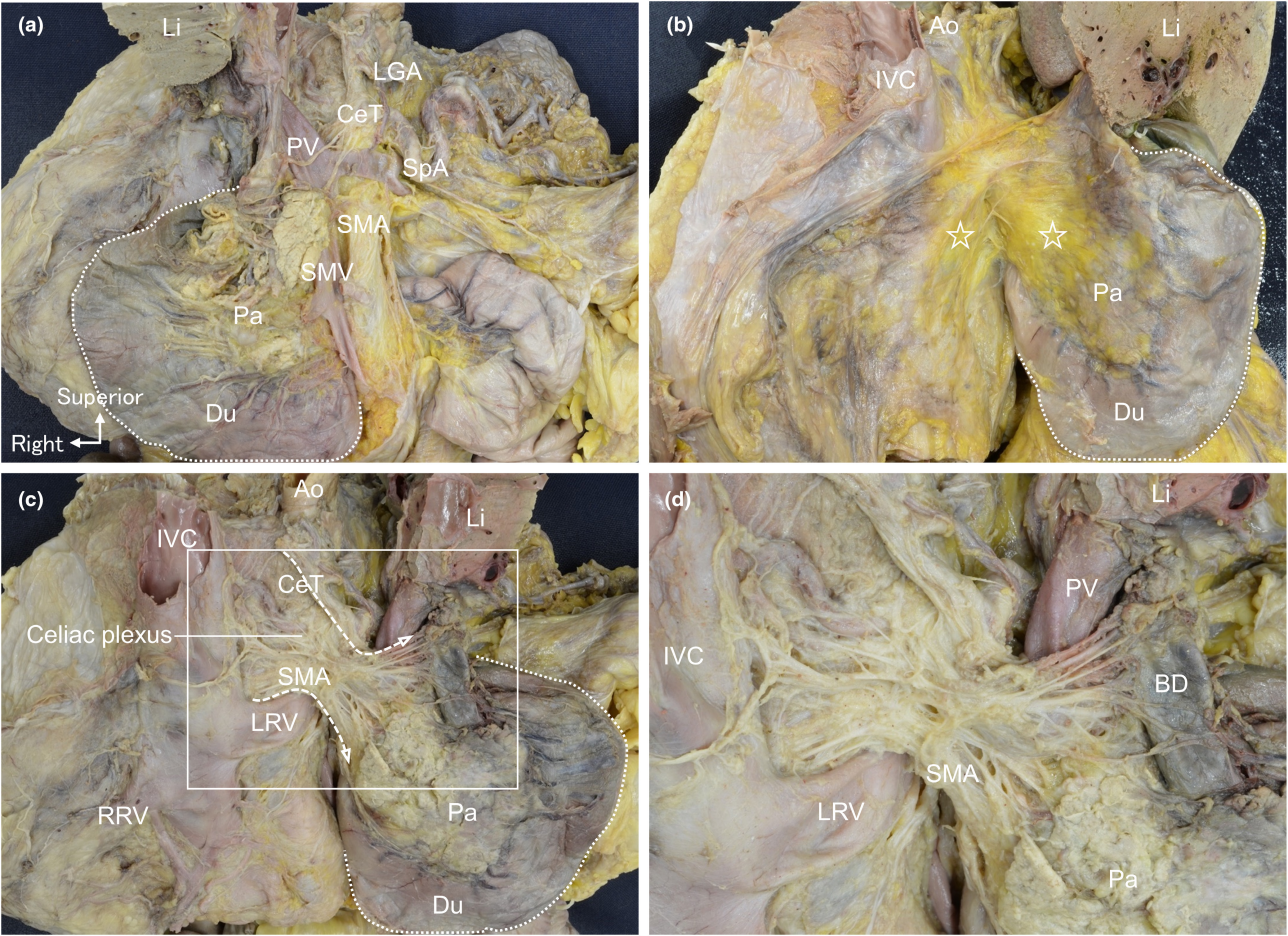
Macroscopic anatomy of the duodenal mobilisation to the medial side and nerve dissection. (a) Ventral view of the pancreatic head and superior mesenteric plexus after removing the pancreatic body and tail. (b) The peritoneum at the right edge of the duodenum (dotted line) is incised, and the duodenum and pancreatic head are mobilised medially. The ventral surface of the retroperitoneal organs and the dorsal surface of the pancreatic head are covered with the fascia (☆). (c) After removal of the fascia, nerves from the celiac plexus converge around the roots of the celiac trunk (CeT) and superior mesenteric artery (SMA), positioning superior to the left renal vein (LRV) (dotted arrow). (d) A magnified view of the white rectangular space from (c). Ao, aorta; BD, bile duct; CeT, celiac trunk; Du, duodenum; IVC, inferior vena cava; LGA, left gastric artery; Li, liver; LRV, left renal vein; Pa, pancreas; PV, portal vein; RRV, right renal vein; SMA, superior mesenteric artery; SMV, superior mesenteric vein; SpA, splenic artery.

We explored the anatomical relationship between the fascia and nerves originating from the dorsal aspect of the pancreatic head. After removing the ventral aspect (body and tail) of the pancreas, the SMA plexus became visible (Figure 1a). The medial aspect of the duodenum and pancreatic head were mobilised alongside the roots of the CeT and SMA, preserving nerves or vessels (Figure 1b). A fascia covered the dorsal surface of the pancreatic head and the ventral side of retroperitoneal organs (stars in Figure 1b). The fascia remained impervious to nerve and vessel penetration. Upon fascia removal, celiac plexus nerves followed the fascia's course and converged around the CeT and SMA roots superior to the left renal vein (Figure 1c). After the convergence, the nerves dispersed towards the dorsal side of the pancreatic head and SMA plexus (Figure 1d).

**FIGURE 2:**
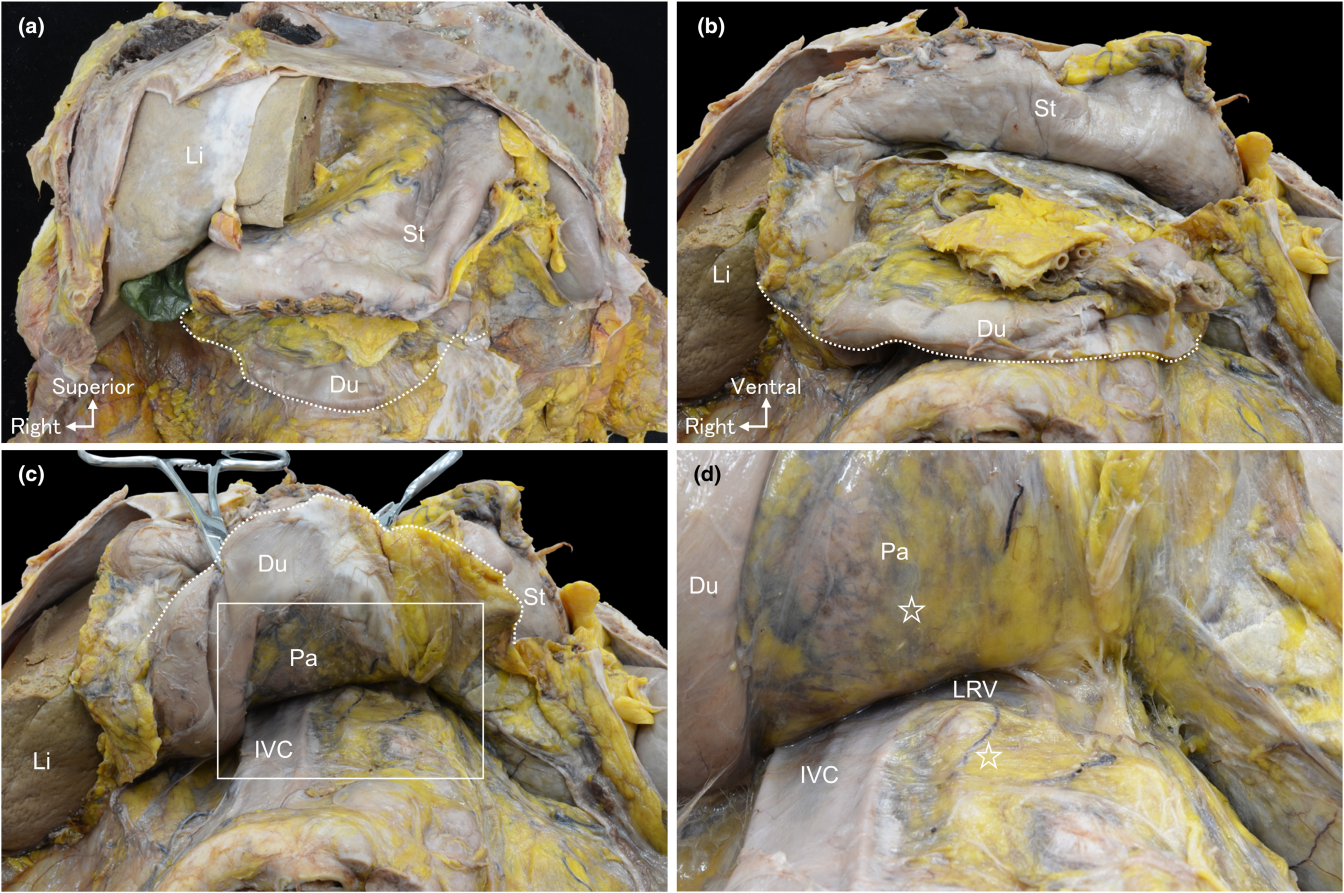
Macroscopic anatomy of the duodenal mobilisation to the cranial side. (a) The ventral side of the upper abdomen following anterior abdominal wall removal. (b) The peritoneum at the inferior edge of the duodenum (dotted line) is incised. (c) The duodenum and the head of the pancreas are mobilised to the cranial side. (d) A magnified view of the white rectangular space in (c) reveals that mobilisation reaches the superior edge of the left renal vein (LRV). The dorsal surface of the head of the pancreas and the ventral surface of the retroperitoneal organs are covered with the fascia (☆). No evident nerves or vessels are observed penetrating through this fascia, visible to the naked eye between the superior border of the LRV and the inferior border of the duodenum. Du, duodenum; IVC, inferior vena cava; Li, liver; LRV, left renal vein; Pa, pancreas; St, stomach.

To examine the distribution of the fascia inferior to the root of SMA, duodenal mobilisation to the cranial side within the same layer was performed as the duodenum and pancreatic head had already been mobilised medially up to the roots of the CeT and SMA (Figure 2a–c). The mobilisation reached the superior border of the left renal vein near the SMA root (Figure 2d). A fascia covered the dorsal surface of the pancreatic head and the ventral aspect of retroperitoneal organs (stars in Figure 2d). No visible nerves or vessels were observed penetrating the dissection plane between the superior border of the left renal vein and the inferior border of the duodenum.

**FIGURE 3:**
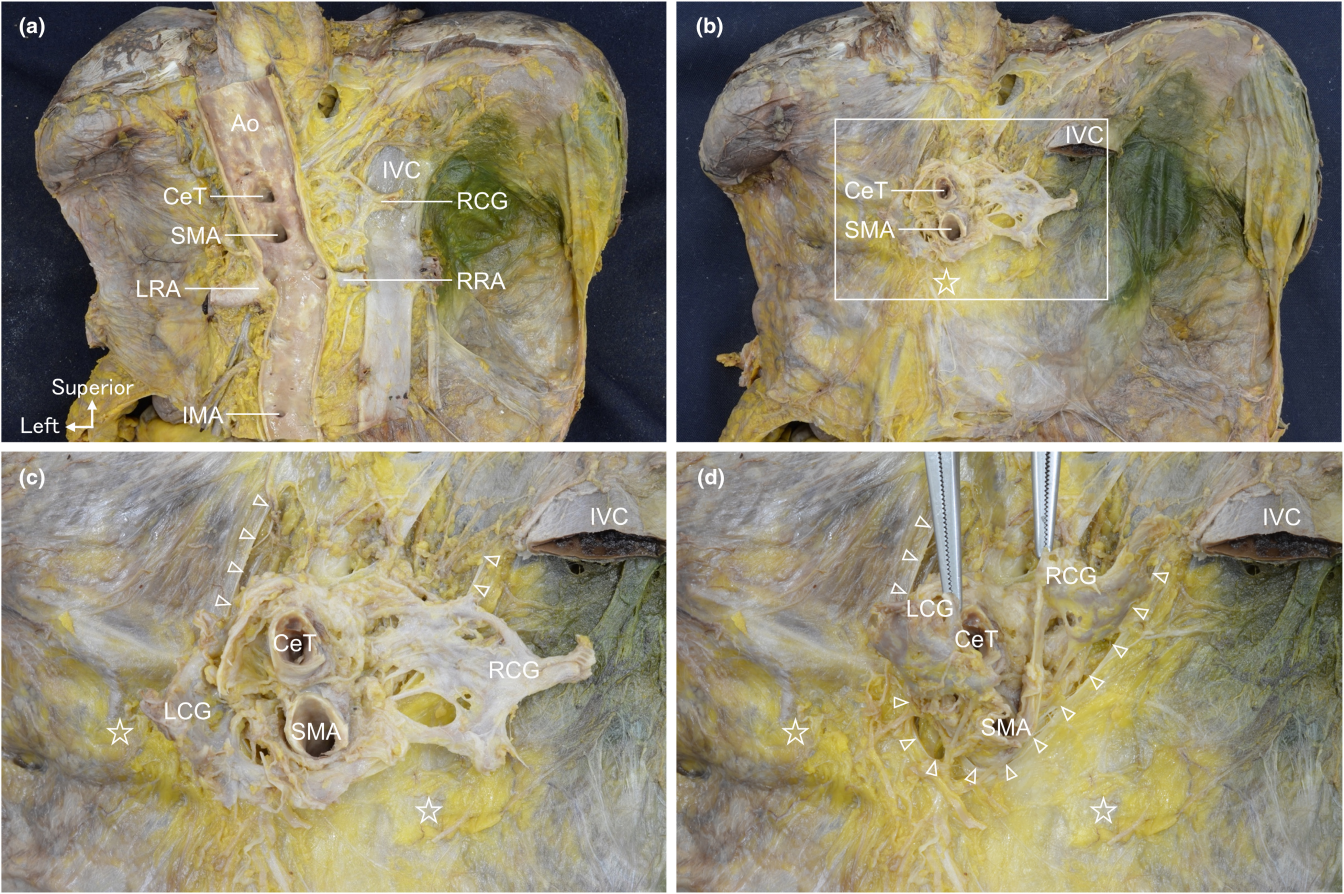
Macroscopic anatomy of the celiac plexus from the dorsal aspect. (a) Dorsal view of the upper abdomen subsequent to removing the aortic posterior wall. The roots of the celiac trunk (CeT), superior mesenteric artery (SMA), and inferior mesenteric artery (IMA) are also observed. The celiac plexus is situated ventral to the aorta and dorsal to the inferior vena cava (IVC). (b) Upon removal of the anterior wall of the aorta and IVC, the complete celiac plexus is exposed, and the fascia (☆) spreads ventrally to the celiac plexus. (c) A magnified view of the white rectangular space in (b). (d) Through the medial and superior reflection of the celiac plexus, the fascia does not cover the roots of CeT and SMA, resulting in a gap much narrower than the celiac plexus (▵). Ao, aorta; CeT, celiac trunk; IMA, inferior mesenteric artery; IVC, inferior vena cava; LCG, left celiac ganglion; LRA, left renal artery; RCG, right celiac ganglion; RRA, right renal artery; SMA, superior mesenteric artery.

After dissecting from the dorsal side and removing the posterior wall of the aorta, the roots of CeT, SMA, and inferior mesenteric artery (IMA) were revealed (Figure 3a). Ventral to the aorta and dorsal to the IVC, a segment of the celiac plexus came into view. Subsequent removal of the anterior wall of the aorta and IVC exposed the entire celiac plexus. The fascia extended ventrally over the celiac plexus and dorsally over the pancreas (star in Figure 3b). Additionally, it enveloped the aorta's anterior surface between the SMA and IMA roots, with no arterial penetration. The IMA descended within the retroperitoneal space and did not extend to the peritoneal organs of the upper abdomen. The celiac plexus exhibited substantial spreading toward both the right and left sides (Figure 3c). Upon medially and superiorly shifting the celiac plexus, it became evident that the fascia did not cover the CeT and SMA root areas, creating a gap connecting the retroperitoneal space and peritoneal organs (arrowheads in Figure 3d). Notably, the celiac plexus's transverse diameter exceeded the no‐fascia area's dimensions (Figure 3c,d). Consequently, nerves from the celiac plexus converge and traverse through the no‐fascia area toward the peritoneal organs.

### 3.2 Histology

**FIGURE 4:**
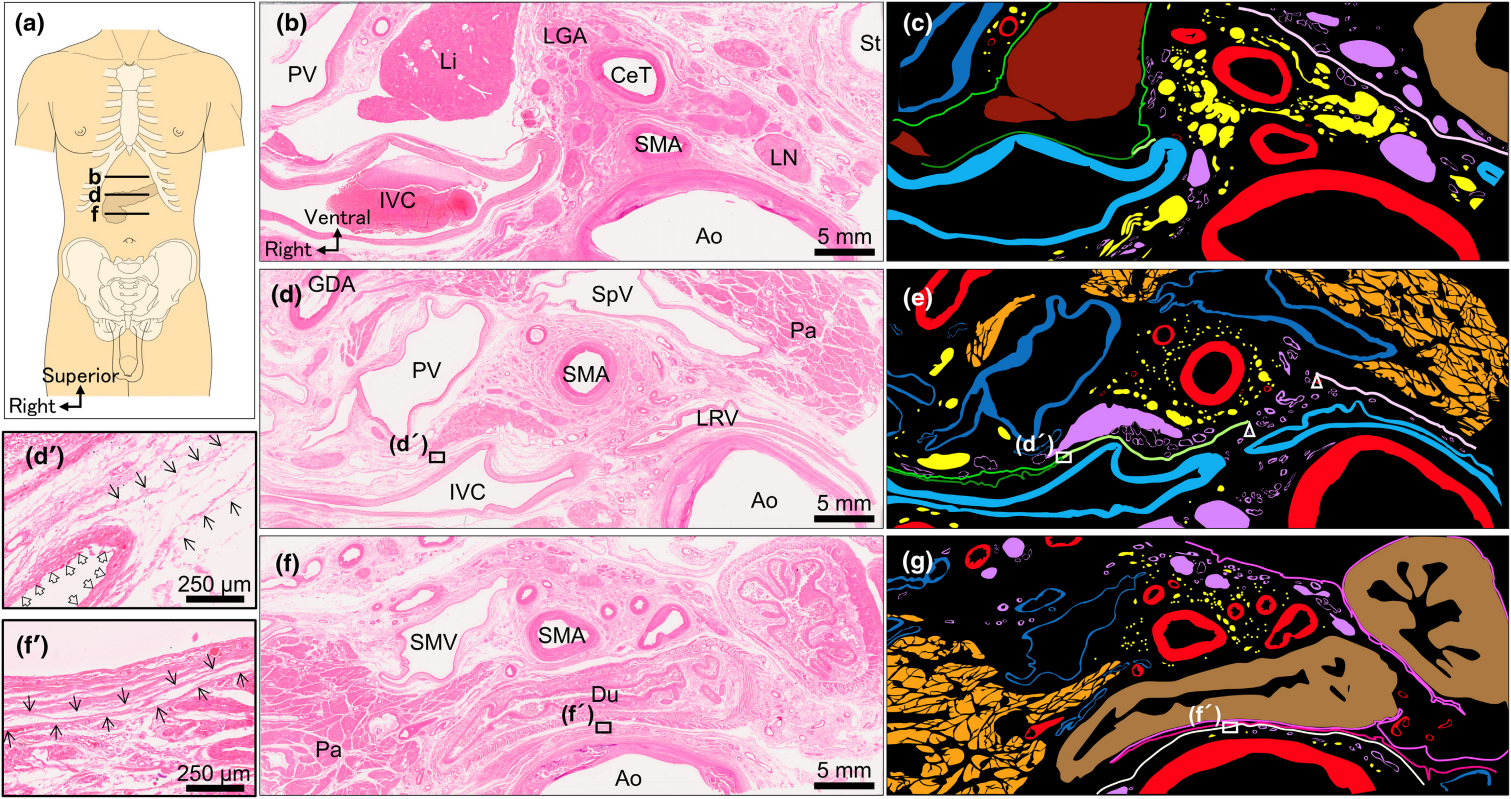
Histological examination of the fascia with Haematoxylin‐Eosin staining. (a) Indicates the section level of (b), (d), and (f). (b) Transverse histological section at the superior mesenteric artery (SMA) root level. (c) The visceral and parietal peritoneum, comprising a simple squamous epithelium, are situated around the liver on the right side of the aorta (green and dark‐green lines). On the left side, there are distinctive layers of collagen fibres (pink line). Arteries and nerves from the retroperitoneal space course through between them. (d) Section at the level of the body and tail of the pancreas. (d´) Magnified view of the black rectangular space in (d). The collagen fibres (lined arrow) area is continuous with the folding point of the simple squamous epithelium (hollow arrow). (e) Several collagen fibres (yellow‐green and pink lines) are situated between the retroperitoneal space and peritoneal organs. Vessels course through between the divided collagen fibre portions (▵). (f) Section at the level of the uncinate process of the pancreas. (f´) Magnified view of the black rectangular space in (f). The layers of collagen fibres are observed (lined arrow). (g) The boundary between the right and left collagen fibres has disappeared dorsal to the duodenum (white line). Ao, aorta; CeT, celiac trunk; Du, duodenum; GDA, gastroduodenal artery; IVC, inferior vena cava; LGA, left gastric artery; Li, liver; LN, lymph node; LRV, left renal vein; Pa, pancreas; PV, portal vein; SMA, superior mesenteric artery; SMV, superior mesenteric vein; SpV, splenic vein; St, stomach.

To delve into the histological attributes of the fascia observed macroscopically, we conducted a wide‐range serial transverse histological sectioning at 0.25‐mm intervals and examined all sections under a microscope. At the SMA root level (Figure 4a,b), we observed the visceral and parietal peritoneum, comprising a simple squamous epithelium around the liver on the right side of the aorta (green and dark‐green lines in Figure 4c). On the left side, distinctive layers of collagen fibres (pink line in Figure 4c) were present. Arteries, including CeT and SMA, and nerves from the retroperitoneal space coursed between the peritoneum and collagen fibres. At the level of the body and tail of the pancreas (Figure 4d), distinct layers of collagen fibres continuous with the peritoneal folding point were visible between the IVC or aorta and the portal vein or pancreas on both the right and left sides (yellow‐green and pink lines in Figure 4e). These fibres marked the boundary between the retroperitoneal space and peritoneal organs. Vessels coursed between the divided collagen fibres portions (arrowheads in Figure 4e). This region of collagen fibres aligned with the macroscopic fascia observation. Additionally, at the uncinate process level of the pancreas (Figure 4f), the boundary between the right and left collagen fibres became obscuring dorsal to the duodenum (indicated by a white line in Figure 4g). Serial sections revealed that the right and left collagen fibres got closer to each other towards the caudal side and boundary between them disappeared on the dorsal side of the duodenum. Nerves and vessels did not traverse the collagen fibres but followed the gap between the two fibre sets.

**FIGURE 5:**
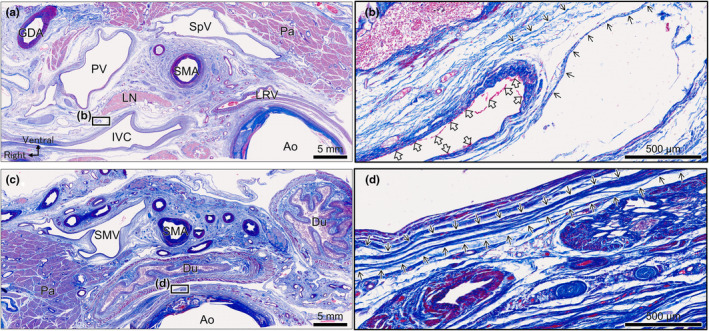
Histological examination of the fascia with Masson's trichrome staining. (a) Section at the level of the body and tail of the pancreas with Masson's trichrome staining. (b) A magnified view of the black rectangular space in (a). The collagen fibres (lined arrow) area is continuous with the folding point of the simple squamous epithelium (hollow arrow). (c) Section at the level of the uncinate process of the pancreas. (d) A magnified view of the black rectangular space in (c). Distinct layers of collagen fibres are observed (lined arrow). The boundary between the right and left collagen fibres is obscured. Ao, aorta; Du, duodenum; GDA, gastroduodenal artery; IVC, inferior vena cava; LN, lymph node; LRV, left renal vein; Pa, pancreas; PV, portal vein; SMA, superior mesenteric artery; SMV, superior mesenteric vein; SpV, splenic vein.

In Masson's trichrome‐stained histological section at the same level as Figure 4d, the collagen fibres were stained blue (Figure 5a). Magnification revealed distinct layers of collagen fibres (lined arrow) that were continuous with the peritoneal folding point (Figure 5b hollow arrow). The levels were similar between Figures 4f and 5c and we analysed 120 slides at 0.25‐mm intervals between Figure 5a,c. We examined whole sections continuously by microscopy, and collagen fibres ran continuously with the fibres in Figure 5b based on their orientation and relationships with surrounding structures. Collagen fibres were identified in the area indicated by lined arrows in the magnified view of the section shown in Figure 5c (Figure 5d). No nerves or vessels penetrated them.

### 3.3 3D reconstruction

**FIGURE 6:**
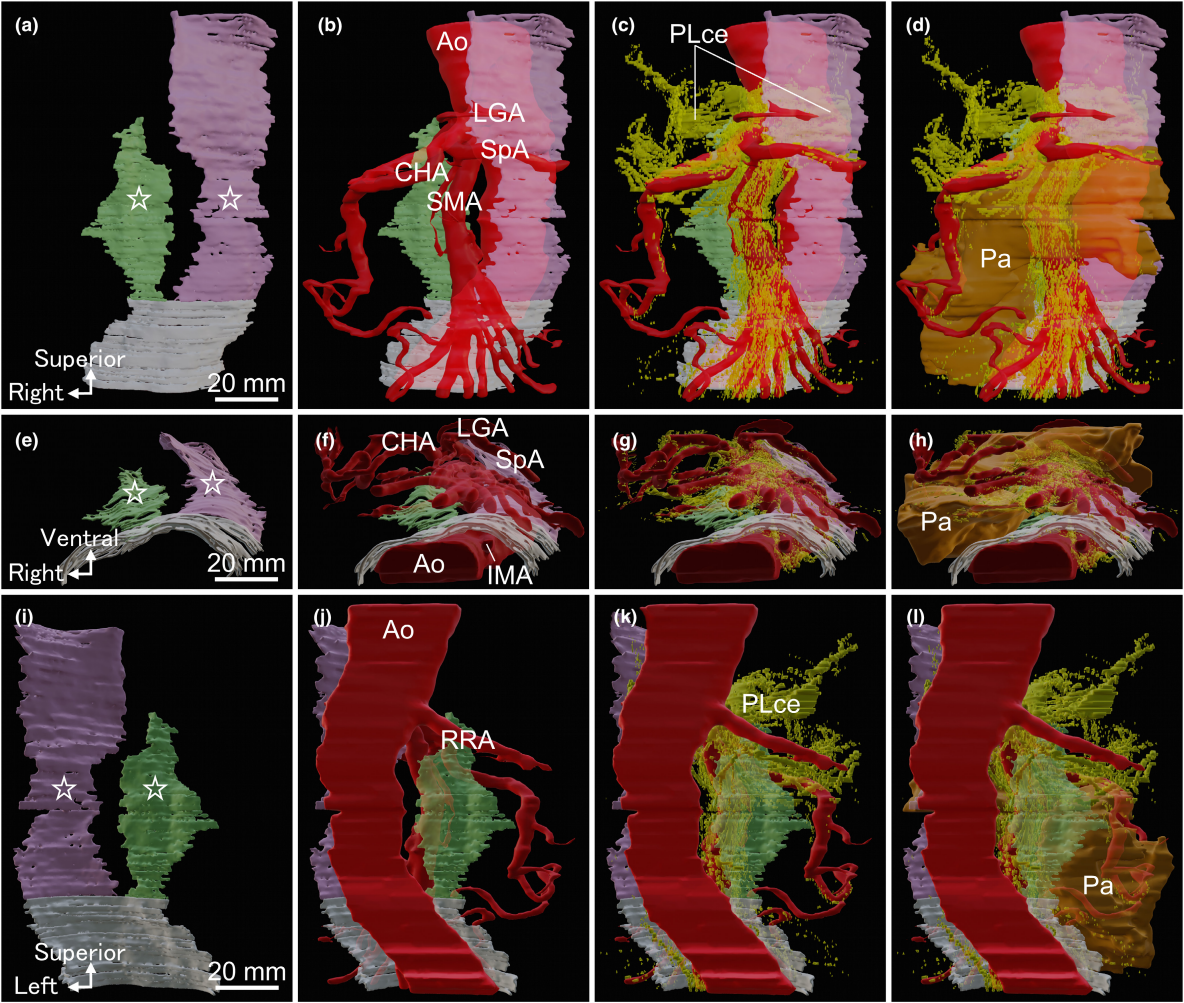
Three‐dimensional (3D) composite image encompassing parts of the sheets formed by collagen fibres corresponding to fasciae of Treitz and Toldt, arteries, nerves, and pancreas (Simple squamous epithelium corresponding to peritoneum and mesentery are not shown). (a) Ventral view of the 3D reconstructed image depicting sheets formed by collagen fibres. The right (yellow‐green) and left (pink) sheets come together and their boundary disappear (white sheet). (b) 3D image of (a) with arteries added. The Celiac trunk (CeT) and superior mesenteric artery (SMA) pass through the gap between the right and left sheets. (c) 3D image of (b) with nerves added. The celiac plexus (PLce) converges and traverses through the gap before branching out towards the pancreas. (d) 3D image of (C) with pancreas added. (e)–(h) Caudal view of the 3D image. (i)–(l) Dorsal view of the 3D image. Ao, aorta; CHA, common hepatic artery; IMA, inferior mesenteric artery; LGA, left gastric artery; Pa, pancreas; PLce, celiac plexus; RRA, right renal artery; SMA, superior mesenteric artery; SpA, splenic artery.

The 3D composite image created from serial sections encompassing collagen fibres, simple squamous epithelium, nerves, arteries, veins, lymph nodes, and pancreas (Supporting Information [Link], [Link]) disclosed sheets formed by collagen fibres (Figure 6a). Positioned ventral to the aorta and dorsal to the pancreas, right and left collagenous sheets (stars in Figure 6a) allowed the passage of CeT and SMA roots through the gaps between them (Figure 6b). These sheets converged caudally at the SMA root. In the caudal part of the serial sections, the collagen fibres that obscured the boundary between the right and left collagen fibres were coloured white. Therefore, the white sheet in Figure 6 was the single entity of the right and left sheets and covered the anterior surface of the aorta inferior to the SMA root. Although the CeT and SMA originated from the retroperitoneal aorta, they did not breach the sheets but instead traversed through the gap between the right and left sheets. Arteries diverged ventrally to the sheets before radiating towards the right and left sides of the peritoneal organs. Additionally, the celiac plexus flanking the aorta converged and transitioned through the gap between the right and left sheets (Figure 6c). Below the sheets, nerves co‐travelled with arteries, radiating towards the pancreatic head. Throughout the pancreatic body, multiple nerves accompany the distribution of splenic arteries. No arteries and nerves breached the collagenous sheets.

## 4 DISCUSSION

**FIGURE 7:**
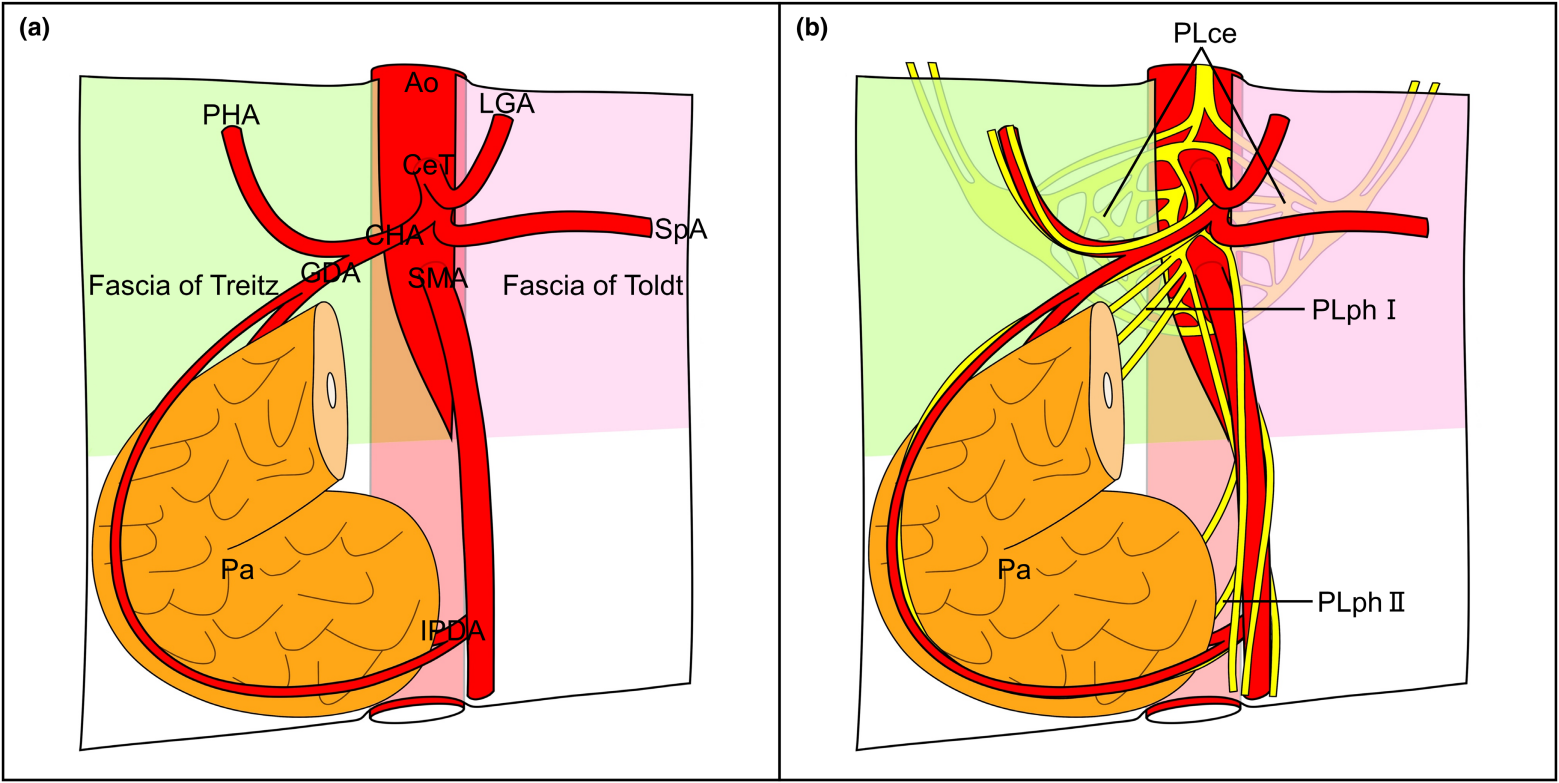
Schematic diagram of the fascia, arteries, and nerves around the pancreas. (a) The fasciae of Treitz and Toldt get closer to each other caudally to the root of the superior mesenteric artery (SMA) and form a narrow gap around the roots of the celiac trunk (CeT) and SMA. (b) The celiac plexus (PLce) does not penetrate the fascia but converges once to traverse through the gap before extending to the pancreas while accompanying the arteries. Ao, aorta; CeT, celiac trunk; CHA, common hepatic artery; GDA, gastroduodenal artery; IPDA, inferior pancreaticoduodenal artery; LGA, left gastric artery; Pa, pancreas; PHA, proper hepatic artery; PLce, celiac plexus; PLphI, pancreatic head plexus I; PLphII, pancreatic head plexus II; SMA, superior mesenteric artery; SpA, splenic artery.

The celiac plexus contains abundant sympathetic and parasympathetic nerve fibres from the coronal, right, and left spinal regions. Positioned broadly on the anterior, right, and left aspects of the aorta at the root of the CeT, it converges before aligning with substantial arteries before spreading to the dorsal surface of the pancreas. The narrower space created by the right and left fasciae around the CeT and SMA roots contrasts with the celiac plexus's convergence point, allowing for clear differentiation of the plexus based on the presence of fasciae (Figure 7).

Previous studies have established that nerves distributed to the pancreatic head and uncinate process originate from the celiac plexus and superior mesenteric plexus, recognised as pancreatic head plexuses I and II (Yoshioka & Wakabayashi, 1958). Our findings indicate that these nerves predominantly accompany the arteries, radiating partly towards the pancreatic head's posterior aspect, consistent with the results of previous studies (Muro et al., 2021; Nagakawa et al., 2020; Yi et al., 2003; Yi et al., 2020; Yoshioka & Wakabayashi, 1958). Additionally, celiac plexus nerves extend radially towards the pancreatic body, primarily alongside the splenic artery. Although earlier studies suggested the entirety of the celiac plexus as the origin of pancreatic nerves, our study reveals the nerves stem solely from the plexus's convergence point around the CeT and SMA roots. The celiac plexus's convergence point contrasts with the narrow space created by the right and left sheet structures comprised of layers of collagen fibres. Hence, the sheet structure divided the nerves from the celiac plexus to the dorsal surface of the pancreas into the retroperitoneal space and peritoneal organ parts. The morphology of the nerves around the pancreas resembles an hourglass, with the sheets distinctly separating the celiac and pancreatic head plexuses.

The layers of collagen fibres comprising the sheets accompanied the folding point of the simple squamous epithelium in the serial histological sections. The position of these simple squamous epithelia relative to organs and arteries indicates that the simple squamous epithelia are the mesentery, visceral, and parietal peritonea. The fusion fascia arises from the amalgamation of the mesentery and parietal peritoneum (Congdon et al., 1942; Toldt, 1893). Therefore, the collagen fibre layers accompanied by the mesentery and peritoneum folding point seem to correspond to the fusion fascia. However, the development of the fusion fascia was often discussed. Previous reports suggest that it was not a simple categorisation as peritoneal remnants (Cho et al., 2009) and that whether there was no fusion fascia in the abdomen (Chen et al., 2023) remained unclear. This study determined the presence of the fascial structure, characterised by distinct layers of collagen fibres in a specific pattern, which did not penetrate nerves and arteries. This was achieved through an extensive 8.5‐cm wide‐range serial histological analysis with 0.25‐mm intervals. Consequently, these fascial structures, acting as the avascular plane between the retroperitoneal and abdominal regions, can be identified as the structures known as the fusion fasciae. Referred to as the fasciae of Treitz and Toldt, these structures are situated on the head's dorsal side and pancreas body‐tail, respectively (Toldt, 1879; Treitz, 1853). Hence, In our 3D image based on wide‐range serial sectioning, the yellow‐green and pink sheets probably signify the fasciae of Treitz and Toldt, respectively. The white sheet in the 3D image seems to represent the part common to both fasciae, and it can form because no arteries or nerves connect the retroperitoneal space and peritoneal organs between the SMA and IMA. This results in an avascular plane being located anterior to the aorta.

The peritoneum's structural composition plays a pivotal role in determining the surgical dissection plane due to the intricate course of nerves and arteries within the mesentery. Recent research has underscored the continuous nature of the mesentery even after fusion, resulting in the fusion fascia (Byrnes et al., 2021; Coffey et al., 2022). This insight implies that dissection along the fascia, coupled with mobilisation, can effectively restore the intestine's original positioning, predating midgut rotation. The Kocher manoeuvre performed during pancreaticoduodenectomy involves dissection along the fascia of Treitz, enabling the mobilisation of the duodenum and pancreatic head to the left side. However, this technique is limited to the first and proximal second segments of the duodenum and pancreatic head due to the attachment of the transverse mesocolon over the second duodenal part (Kocher., 1903; Mirilas & Skandalakis, 2010). However, the duodenal mobilisation to the cranial side after the Cattell–Braasch manoeuvre performed during pancreaticoduodenectomy offers the advantage of mobilising the entire duodenum (Cattell & Braasch, 1960; Mirilas & Skandalakis, 2010). This manoeuvre facilitates the exposure of the SMA's origin as a portion of the duodenum traverses the dorsal aspect of the artery (Del Chiaro et al., 2015). The membranous structure that connects to the dorsal duodenum and pancreatic head anterior to the aorta post the duodenal mobilisation to the cranial side is suggested to result from the common part of Treitz and Toldt's fasciae.

The precise analysis of fascia distribution revealed a confined connection between the retroperitoneal space and peritoneal organs, primarily centred around the CeT and SMA roots. Notably, nerves did not breach the fascia but navigated through a narrow gap covered by it. Similarly, arteries originating from the retroperitoneal aorta and extending to the peritoneal organs traversed this gap. Consequently, this study's significance lies in showcasing the tight passage between the retroperitoneal space and peritoneal organs, offering insights into intraoperative nervous and vascular anatomy.

This study has some limitations. First, the extent of variation in the gap between fasciae remains unexplored. However, this gap can be addressed by amalgamating diverse approaches, including macroscopic anatomy, histological analysis, and 3D reconstruction from the eight cadavers. Second, the donors of the cadavers used in our study were primarily older adults, with an average age of >80 years, due to the unavailability of cadavers from younger adults. This skewed age distribution, distinct from typical clinical patient populations, introduces potential impacts of ageing that must be acknowledged.

## 5 CONCLUSION

Although not directly connecting with the intra‐abdominal organs, the celiac plexus converges within a slender gap within the fascia before its subsequent dispersion. The distinctive nerve morphology observed, resembling an hourglass, arises from the convergence of the celiac plexus through the gap between the fasciae on both sides, eventually fanning out toward the pancreas. The right and left side fasciae get closer to each other and cover the aorta's anterior surface between the SMA and IMA roots. Our study underscores that the retroperitoneal space and peritoneal organs are bridged solely by a narrow gap between the CeT and SMA, along with the nerves enveloping these arteries. This anatomical insight constitutes a foundational understanding of the course of nerves and vasculature pertinent to abdominal digestive organ surgery.

## AUTHOR CONTRIBUTIONS

Yuzuki Sugiyama contributed to the conception and design of the work; acquisition, analysis, and interpretation of data; drafting of the manuscript; and final approval of the article. Satoru Muro contributed to the conception and design of the work; acquisition, analysis, and interpretation of data; critical revision of the manuscript; and final approval of the article. Daisuke Ban contributed to the analysis and interpretation of data, critical revision of the manuscript, and final approval of the article. Keiichi Akita contributed to the study conception and design of the work, analysis and interpretation of data, critical revision of the manuscript, and final approval of the article.

## Supporting information


**Supporting Information S1.** 3D composite image created from serial sections encompassing the arteries, nerves, pancreas, collagen fibres, simple squamous epithelium, veins, and lymph nodes.

**Supporting Information S2.** Movie of the 3D image.

## Data Availability

Data supporting this study's findings are available from the corresponding author on a reasonable request.
